# Correction of Hypernatremia Due to Pure Dehydration Could Be a Potential Risk Factor for Transient Atrial Fibrillation

**DOI:** 10.7759/cureus.5387

**Published:** 2019-08-14

**Authors:** Sanjay Timilsina, Ramakanth Pata, Sambida Timilsina, Shalini Cherala, Paritosh Kafle

**Affiliations:** 1 Internal Medicine, Interfaith Medical Centre, Brooklyn, USA; 2 Pulmonary Medicine, Interfaith Medical Center, Brooklyn, USA; 3 Internal Medicine, Interfaith Medical Center, Brooklyn, USA

**Keywords:** chronic hypernatremia, atrial fibrillation, dehydration

## Abstract

Although atrial fibrillation (AF) has been correlated with hyponatremia, it has not been described in association with the correction of chronic hypernatremia in previous studies.

Here, we describe three elderly patients who had hypernatremia on presentation, most likely secondary to dehydration. Their electrocardiogram (EKG) on presentation was normal, and they had no known history of AF but developed transient AF during the course of hypernatremia correction. Similar occurrences have not been reported previously though there have been studies showing the relation between hyponatremia and AF. It is known that atrial stretch can increase the propensity of rapid firing from the pulmonary veins as a result of stretch-sensitive ion channels. Therefore, the occurrence of AF in these patients could be explained by the phenomenon of increase in right atrial diameter, i.e atrial stretch, in response to the increase in preload during the course of treatment. However, there are many other clinical scenarios where boluses of fluid are given during treatment but no occurrences of AF have been reported so far. So, we put forward the possibility of the occurrence of AF being independently associated with the drop in sodium level. Further studies like speckle tracking echo might shed more light on these findings.

## Introduction

Hypernatremia is defined as a rise in the serum sodium concentration to a value exceeding 145 mmol per liter. It is a common electrolyte disorder [1]. Hypernatremia due to water depletion/hypodipsia is called dehydration. This differs from hypovolemia, in which both salt and water are lost [2]. The signs and symptoms of acute dehydration are thirst and, progressively, confusion, coma, and respiratory paralysis. Hypernatremia causes a rise in plasma tonicity and stimulates thirst as well as a release of antidiuretic hormone (ADH), thereby minimizing water loss and increasing water intake [1]. In healthy individuals who respond to thirst, this phenomenon brings the serum sodium concentration back to normal. As a result, hypernatremia is seen primarily in patients who cannot experience or respond to thirst normally due to impaired mental status (eg, older adult or critically ill patients) [3-6] and in disabled/demented people who require others to provide fluid intake [1,3]. Chronic hypernatremia (>48 hr) is a common occurrence in patients residing in long-term care facilities, as many such patients are old, often demented, or have a physical condition that might limit access to water. Hypernatremia as a consequence of pure hypodipsia is of gradual onset. The gradual development of hypertonicity lets the brain and other tissues adapt by generating new intracellular solutes (previously called idiogenic osmoles) to minimize shrinkage [3]. Because of the cerebral adaptation, neurologic symptoms are much less severe with chronic hyponatremia. Patients with chronic hyponatremia may appear to be asymptomatic despite a serum sodium concentration that is persistently below 120 mEq/L. Symptoms that do occur include fatigue, nausea, dizziness, gait disturbances, forgetfulness, confusion, lethargy, and muscle cramps. When exacerbated by the pathologic conditions associated with increased fluid loss, patients may present with acute changes. Chronic hypernatremia must be corrected slowly to prevent rapid fluid movement into the brain and the resulting cerebral edema leading to complications like seizures and altered mental status. The correction should be by no more than 12 mEq/L per day.

Atrial fibrillation (AF) is the most common cardiac arrhythmia in elderly persons [7]. There are many clinical situations in the past explaining the onset of AF in relation to other medical conditions like hyperthyroidism, acute pulmonary embolism, myopericarditis, pneumonia, post-cardiac surgery, etc. Factors like old age, diabetes mellitus (DM), hypertension (HTN), dyslipidemia, and chronic kidney disease (CKD) have also been associated with atrial fibrillation. There has also been a study where the presence of increased left atrial (LA) diameter in echocardiography was independently associated with AF [8]. The same study also showed hyponatremia as an independent cause of AF in patients with systolic heart failure [8]. However, there have not been studies showing the occurrence of AF in relation to the correction of chronic hypernatremia or a drop in Na level. Hereby, we have presented three cases who developed transient AF during the course of correction of chronic hypernatremia. The atrial stretch due to the increase in preload during the correction of hypernatremia can possibly be an explanation for these episodes of transient AF, as there were no other obvious precipitating causes, or the occurrence of AF might also be independently related to the drop in sodium level.

## Case presentation

Patient 1

An 80-year-old African American male with a past medical history (PMH) of dementia (non-communicative at baseline, activities of daily living (ADLs) dependent), cerebrovascular accident (CVA) with right-sided hemiplegia and aphasia, diabetes mellitus (DM) type 2, hypertension (HTN), benign prostatic hyperplasia (BPH) was sent to our emergency department (ED) from his nursing home for the management of altered mental state. The patient had decreased food and water intake for the past two weeks. He had no fever, headache, neck stiffness, seizure activity, rashes, abdominal tenderness, vomiting, or diarrhea. Medications were reconciled with nursing home records. These included donepezil, glimepiride, finasteride, terazosin, gabapentin, and aspirin.

On examination, the patient was drowsy. His Glasgow Coma Scale (GCS) was 11/15; he was tachycardic with a pulse of 124/min, tachypneic with a respiratory rate (RR) of 28/min, blood pressure (BP) was 106/64 mm of Hg, temperature was 99.7 F, and oxygen saturation was 98% at room air. The patient was noted to have dry oral mucosa and decreased skin turgor. He also had a stage I decubitus ulcer in the left lateral heel, which looked clean. Signs of meningismus were absent. Except for the preexisting neurological deficits, the rest of the systemic examination was unremarkable (Table 1).

**Table 1:** Admission workup

Item	Value (normal reference range)
Sodium Level	172 mmol/L (135-145 mmol/L)
Potassium Level	3.6 mmol/L (3.5-4.5 mmol/L)
Chloride Level	140 mmol/L (98-106 mmol/L)
Carbon Dioxide Level	21 mmol/L (23-28 mmol/L)
Anion Gap	11 (8-16)
Blood Urea Nitrogen	58 mg/dL (8-20 mg/dl)
Creatinine	2.54 mg/dL (0.7-1.3mg/dl)
Glucose Level	172 mg/dL (130-180mg/dl)
Lactic Acid Level	2.0 mmol/L (0.5-2.2 mmol/L)
Calcium Level	9.0 mg/dL (9-10.5 mmol/L)
White Blood Count	15.1 10^3/uL (4-10 10^3/uL)
Hemoglobin	11.3 g/dl (M: 14-17g/dl, F: 12-16 g/dl)
Hematocrit	35.8% (M: 41-51%, F: 36%-47%)

Initial biochemical evaluation (Table 1) revealed severe hypernatremia and acute kidney injury (AKI) with lactic acidosis. White blood cell (WBC) was 15,000/cu mm. EKG showed normal sinus rhythm. Chest X-ray was normal. Urinalysis was negative for infection. A computed tomography (CT) scan of the head did not reveal any acute changes. Cerebrospinal fluid (CSF) analysis was negative. Echocardiogram was normal with an ejection fraction (EF) of 55%-60%. The patient was managed in the intensive care unit (ICU) with a provisional diagnosis of metabolic encephalopathy secondary to chronic hypernatremia to rule out possible sepsis.

Calculated free water deficit was 7 liter (L). As there was evidence of volume, depletion rehydration was initially considered with isotonic solutions. A 2 L normal saline (NS) bolus was given in the emergency department (ED). Dextrose 5% in normal saline (D5NS) @120ml/hr and 300 ml of free water via a nasogastric tube (NG) tube every four hours were started, and the basic metabolic panel (BMP) was monitored every two hours. After 20 hours, intravenous (IV) hypotonic solutions were started as per the Androgue Madias formula aiming for a 10 meq drop of Na per day [9]. Hypernatremia trended down and the AKI was improving (Table 2). Results of blood cultures and urine culture came out to be negative. As no focus of infection was identified, antibiotics were discontinued. On Day 3, the telemetry monitor showed new-onset paroxysmal atrial fibrillation with a heart rate (HR) of around 100-140 for which metoprolol was given on an as-needed basis along with Eliquis. The thyroid function test was done; the results were within the normal range. From Day 6, the surveillance of the telemetry monitor did not reveal any further AF for the rest of the stay in the hospital. Considering the age and other comorbidities, Eliquis was later discontinued at the request of next of kin (NOK), and the patient was in sinus rhythm since then. Speech therapist evaluation was performed and a percutaneous endoscopic gastrostomy (PEG) tube was placed. The patient was discharged to a nursing home with a loop recorder.

**Table 2:** Follow-up labs BUN: blood urea nitrogen

	Sodium (mmol/L)	BUN (mg/dl)	Creatinine (mg/dl)
Day 1	172	58	2.54
Day 2	169	65	2.34
Day 3	164	48	1.63
Day 4	156	36	1.44
Day 5	149	24	1.32
Day 6	144	23	1.30

Patient 2

An 89-year-old female, with a past medical history of heart failure with preserved ejection fraction (HFpEF), dementia (non-communicative at baseline, activities of daily living (ADL) dependent), HTN, osteoarthritis, gastroesophageal reflux disease (GERD), and recent displaced oblique fracture of the right femoral shaft, was sent to the emergency room from a nursing home facility for altered mental status of one day duration.

At the time of presentation, she was minimally responsive to verbal and tactile stimuli and showing some movement of the upper and lower limbs. She was hypotensive with BP of 85/58. Her temperature was 97.4 F, pulse rate (PR) was 97 b/m, and oxygen saturation was 100% on a non-rebreather oxygen mask. She appeared severely dehydrated. A right lower extremity cast was in place for a displaced oblique fracture of the femoral shaft. She also had a large, unstageable sacral decubitus ulcer. Signs of meningismus were absent. The rest of the examination was unremarkable, and a detailed central nervous system (CNS) examination couldn’t be performed given the circumstances. Lab values at the time of admission were as shown in Table 3.

**Table 3:** Admission workup

Item	Value (Normal ref range)
Sodium Level	168 mmol/L (135-145 mmol/L)
Potassium Level	2.9 mmol/L (3.5-4.5 mmol/L)
Chloride Level	127 mmol/L (98-106 mmol/l)
Carbon Dioxide Level	27 mmol/L (23-28 mmol/L)
Anion Gap	11 (8-16)
Blood Urea Nitrogen	80 mg/dL (8-20 mg/dl)
Creatinine	1.76 mg/dL (0.7-1.3 mg/dl)
Glucose Level	127 mg/dL (130-180 mg/dl)
Lactic Acid Level	2.9 mmol/L (0.5-2.2 mmol/L)
Calcium Level	9.0 mg/dL (9-10.5 mmol/L)
White Blood Count	15.510^3/uL (4-10 10^3 /uL)
Hemoglobin	11.5 g/dl (M: 14-17 g/dl, F: 12-16 g/dl)
Hematocrit	37.4% (M: 41-51%, F: 36-47%)
Platelets	210^3/uL (150-450*10^3/uL)
Urinanalysis	Nitrate positive, Leukocyte esterase negative, Protein trace, Ketones trace, Blood trace

An initial chest X-ray showed mild congestion, with a small, right-sided pleural effusion. A CT scan of the head showed peri-ventricular white matter changes and old lacunar infarcts in the basal ganglia. The CSF analysis was negative. An echocardiogram was also done, which showed normal left ventricular function, with an EF of 55%-60% with an elevated pulmonary artery pressure (PAP) of 45 mmHg. She also had a mechanical mitral prosthesis with mild to moderate mitral regurgitation (MR), with a mean gradient of 7 mmHg across the valve. EKG showed a sinus rhythm with a rate of 72 and occasional premature atrial contractions (PACs).

With these findings, she was admitted to the ICU for acute delirium likely due to severe dehydration, dyselectrolytemia, and possible sepsis. Given the signs of hypovolemia, she was initially treated with normal saline (NS) boluses followed by the treatment of hypernatremia with hypotonic fluids with the aim to correct the sodium to a target level of 10 meq below her presentation over the next 24 hours. Broad-spectrum antibiotics were started for possible sepsis. Blood culture, urinalysis, and urine culture reports came negative. During the course of treatment, hypernatremia trended down as shown in Table 4.

**Table 4:** Trending of sodium

Date	Serum Na value (mmol/l)
Day 1	168
Day 2	164
Day 3	158
Day 4	155
Day 5	152
Day 6	150

During the course of the correction of her hypernatremia, she developed multiple episodes of atrial fibrillation. The thyroid function test was done; results were within the normal range. Her hospital course was complicated further due to multiple comorbidities, and, unfortunately, she succumbed to her illness. HF with mitral valve prosthesis with mild to moderate MR is a potential risk factor for the occurrence of AF. but given the patient's history of no previous incidents of AF and seeing the temporal association with the correction of hypernatremia, we raise a question if this is a possible trigger.

Patient 3

A 66-year-old male with PMH of the CVA with left hemiparesis (with contractures), ADL dependent, and non-communicative at baseline, dysphagia s/p PEG placement, peripheral artery disease (PAD) s/p right above-knee amputation (AKA), coronary artery disease (CAD), DM, chronic obstructive pulmonary disease (COPD), hypertension, and epilepsy was sent from a nursing home for altered mentation and shortness of breath (SOB).

The patient had no cough, rashes, vomiting, or diarrhea. At the time of admission, the patient was febrile with a temperature of 101°F, tachycardic with an HR of 161 b/m. His RR was 16 and BP was 104/77 mmHg. He was drowsy with intermittent hiccups. He had a PEG tube in-situ with a scar near the PEG tube. The PEG tube stoma looked clean, with no signs of inflammation. Signs of meningismus were absent. The rest of the systemic examination was unremarkable except for the preexisting neurological deficits. Initial lab values were as shown in Table 5.

**Table 5:** Admission workup

Item	Value (normal reference range)
Sodium Level	161mmol/L (35-145 mmol/L)
Potassium Level	3.6 mmol/L (3.5-4.5 mmol/L)
Chloride Level	124mmol/L (98-106 mmol/L)
Carbon Dioxide Level	21 mmol/L (23-28 mmol/L)
Anion Gap	11 (8-16)
Blood Urea Nitrogen	31 mg/dL (8-20 mg/dl)
Creatinine	1.22mg/dL (0.7-1.3 mg/dl)
Glucose Level	140mg/dL (130-180 mg/dl)
Lactic Acid Level	3.1mmol/L (0.5-2.2 mmol/L)
Calcium Level	7.7 mg/dL (9-10.5 mmol/L)
White Blood Count	18.8^10^3/uL (4-10 *10^3/uL)
Hemoglobin	18.5g/dl (M: 14-17 g/dl, F: 12-16 g/dl)
Hematocrit	58.1% (M: 41-51%, F: 36-47%)
Urinanalysis	Nitrate negative, Leukocyte esterase negative, RBCs: 50-100, WBCs: 1-5, Protein trace, Ketones trace

The chest X-ray showed no acute changes as compared to the past chest X-rays. CT chest showed changes compatible with COPD, with no acute changes or consolidation. Urinalysis was negative for infection. CT head revealed a chronic subdural hematoma over the right cerebral hemisphere with no mass effect and no change in size as compared to the past CT head. Lumbar puncture (LP) was deferred. She was admitted to the intensive care unit (ICU) for an altered mental state due to possible sepsis and chronic hypernatremia. Antibiotics were started. The calculated free water deficit was 5.2 liters. As there was evidence of volume depletion, rehydration was initially considered with isotonic solutions. A 2 L NS bolus was given in the emergency department (ED), NS @80ml/hr. BMP was monitored every two hours. After 24 hours, IV hypotonic solutions was started as per the Androgue Madias formula aiming for 10 meq drop of Na per day. Blood culture and urine cultures showed no growth. Hypernatremia trended down gradually over five days, as shown in Table 6. The patient became medically stable and more aware and responsive.

**Table 6:** Sodium level trend

Date	Serum Na value (mmol/l)
Day 1	161
Day 2	159
Day 3	156
Day 4	154
Day 5	144

On Day 4 of correction of hypernatremia and dehydration, the patient remained tachycardic and developed a few episodes of atrial fibrillation. Thyroid function test (TFT) was done. The results of the TFT were within the normal range. No more episodes of AF were noted, as hypernatremia resolved.

## Discussion

Hypernatremia is a common occurrence in the nursing home setting, as many of the patients are often old and/or demented or have a physical condition that might limit access to water. This condition is often exacerbated by pathological conditions like acute infections with increased fluid loss. Furthermore, older adult patients also appear to have diminished osmotic stimulation of thirst [5-6]. Hypernatremia, as a consequence of pure hypodipsia, is gradual in onset, causing a very subtle change in cognition, which is usually missed unless critical cognitive tests are done. Here, we have presented three case scenarios with chronic hypernatremia in whom episodes of transient atrial fibrillation incurred during the course of correction of hypernatremia. Though, on admission, the provisional diagnosis also included sepsis, no obvious focus of infection was identified with labs, microbiology, and imaging. Moreover, these patients had no prior history of AF despite the risk factors like old age, DM, HTN, COPD, and CAD that have been associated with AF. Given the absence of a past history of the occurrence of AF and accounting for the temporal association with the correction of hypernatremia in three similar scenarios, we are raising a question on the correction of hypernatremia being a possible trigger.

It is known that atrial stretch can increase the propensity for rapid firing from the pulmonary veins as a result of stretch-sensitive ion channels [10]. It has also been speculated that the mechanism of atrial stretch may help explain the association between AF and mitral regurgitation as well as various types of heart failure [8]. The occurrence of atrial fibrillation in these patients may be explained by the phenomenon of an increase in right atrial diameter, i.e. atrial stretch in response to the increase in preload during the course of treatment. There are other clinical scenarios where boluses of fluid are given, resulting in increased preload and, therefore, atrial stretch, and, yet, no events of AF have so far been reported. We hereby put forward the possibility of AF being independently associated with the drop in sodium level.

## Conclusions

Atrial fibrillation has not been previously described in association with the correction of chronic hypernatremia, although atrial fibrillation has been correlated with hyponatremia. The incidence of transient atrial fibrillation in patients with chronic hypernatremia in the course of correction of Na+ levels is not well-understood. In the future, more studies like speckled track echo are needed to establish the relation and understand the phenomenon better. The aim of this case series is to give recognition to a possible phenomenon that has not been previously reported; a deeper exploration into this might lead to valuable findings.
